# Assessing and Improving the Documentation of Large-Volume Paracentesis Procedures in a Tertiary Hospital: A Quality Improvement Project

**DOI:** 10.7759/cureus.74796

**Published:** 2024-11-29

**Authors:** Aravind Sunderavel Kumaravel Kanagavelu, Ashweta Josan, Rimsha Khan, Israa Alhammo, Elham Barati, Vikram Sharma

**Affiliations:** 1 Gastroenterology and Hepatology, Barts Health NHS Trust, London, GBR

**Keywords:** accuracy of documentation, ascitic drain, large volume paracentesis, patient safety improvement, quality improvement projects

## Abstract

Inconsistent documentation of large-volume paracentesis (LVP) procedures in a tertiary hospital presents risks to patient safety and procedural quality. This study aimed to improve the completeness and accuracy of LVP documentation through the implementation of a structured checklist, developed in alignment with the British Society of Gastroenterology (BSG) Safety Toolkit. The intervention was conducted over three Plan-Do-Study-Act (PDSA) cycles and involved multidisciplinary collaboration, the integration of Local Safety Standards for Invasive Procedures (LocSSIPs) into the Clinical Record System (CRS), and targeted training for staff. Sample sizes across the cycles were 35, 34, and 35 participants, respectively. Significant improvements were achieved in key documentation metrics, encompassing pre-procedure, post-procedure by doctors, and post-procedure by nurses. The results demonstrate the efficacy of structured interventions in standardising procedural documentation, improving compliance, and enhancing patient safety within clinical practice.

## Introduction

Cirrhosis of the liver, characterised by scarring due to various aetiologies, is a chronic and progressive condition with significant morbidity and mortality. The most common causes of cirrhosis are alcohol-related liver disease and viral hepatitis [1]. Cirrhosis leads to multiple complications, among which ascites is one of the most common. Ascites are a pathological accumulation of fluid in the peritoneal cavity, typically resulting from portal hypertension and the dysregulation of sodium and water retention mechanisms [1].

The first-line treatment for ascites involves diuretics, such as spironolactone and furosemide [1]. However, in cases of diuretic-resistant or intractable ascites, or when fluid accumulation recurs rapidly, large-volume paracentesis (LVP) is required [2]. This procedure involves the removal of large amounts of ascitic fluid and aims to alleviate symptoms such as abdominal discomfort and respiratory compromise. It does not, however, influence the progression of the underlying disease [1]. For patients with frequent fluid re-accumulation, transjugular intrahepatic portosystemic shunt (TIPS) placement may be considered to reduce portal pressure [1]. Ultimately, liver transplantation remains the only curative treatment for cirrhosis [3].

Like any invasive procedure, LVP carries potential risks, including pain, spontaneous bacterial peritonitis, haemorrhage, and bowel perforation; however, these complications are rare, occurring in fewer than 1 in 1,000 cases [2]. To mitigate risks, baseline laboratory tests, such as platelet counts and international normalised ratio (INR), are recommended. A platelet count above 50 × 10⁹/L and an INR below 2 are generally targeted for optimal procedural safety [2]. While no absolute contraindications exist, prophylactic administration of blood products may be considered to improve outcomes and minimise complications [1]. For patients requiring the drainage of more than 5 L of ascitic fluid, albumin replacement with human albumin solution (HAS) is recommended to prevent post-paracentesis circulatory dysfunction (PPCD) and renal impairment [2]. The standard replacement protocol involves administering 100 mL of 20% HAS for every 2-2.5 L of ascitic fluid removed [1]. Thus, despite being a common procedure, LVP requires meticulous attention to detail to ensure safe and effective execution by both doctors and nursing staff.

At our hospital, approximately 15-20 LVP procedures are performed monthly across the hepatology ward and the ascitic drain outpatient clinic. Observations revealed considerable variability in documentation practices among healthcare professionals, highlighting the need for a standardised approach. Inconsistent records can compromise patient safety, procedural accuracy, and continuity of care, necessitating the development of a user-friendly checklist to address these issues [4].

In response, we initiated a quality improvement project (QIP) using the Plan-Do-Study-Act (PDSA) cycle, a tested and proven methodology in quality improvement initiatives [5]. Initial data confirmed significant disparities in documentation practices, prompting multiple iterative interventions. Using the British Society of Gastroenterology (BSG) Safety Toolkit as a foundation, we developed and implemented a comprehensive, structured checklist for LVP documentation [6]. This checklist aimed to enhance the accuracy and completeness of records across three critical domains: pre-procedure documentation by doctors, post-procedure documentation by doctors, and post-procedure documentation by nurses. This study evaluates the impact of this intervention and its role in promoting safer and more consistent practices in the management of ascites.

## Materials and methods

Setting

The study was conducted in the Department of Hepatology at a tertiary care hospital over a 12-month period, from August 2023 to July 2024. A multidisciplinary team of doctors and nurses participated, focusing on LVP procedures and the care of patients requiring them. Both inpatient and outpatient services, including those provided by the ascitic drain clinic, were included. Procedures deemed unsuccessful or those performed in other departments - such as the intensive care unit, surgical units, or acute medical units - were excluded from the study.

Baseline assessment

Initial discussions with consultant hepatologists revealed the absence of pre-existing local guidelines or procedural documentation specific to LVP. To establish a baseline, a retrospective review of documentation from 35 LVP procedures performed between August and September 2023 was undertaken. This assessment identified significant variability in compliance with key metrics when compared to the BSG Safety Toolkit. Compliance rates ranged from 0% for monitoring pain or haematoma at the drain site to 100% for ensuring the availability of HAS. Furthermore, notable discrepancies in documentation were observed between different doctors and nurses, underscoring the need for a structured and standardised approach to LVP procedure documentation.

Implementation and intervention

Three PDSA cycles were conducted with sample sizes of 35, 34, and 35 participants, respectively, over the study period as follows:

Cycle 1 (August-November 2023)

Initial data collection was followed by the creation and implementation of a checklist. To ensure essential details were recorded systematically, a Local Safety Standards for Invasive Procedures (LocSSIPs) checklist was developed in alignment with the BSG Safety Toolkit and designed according to National Safety Standards for Invasive Procedures 2 (NatSSIPs 2) guidelines (Table 1). This procedural note was validated by hepatology consultants and integrated into the Clinical Record System (CRS). Individual training sessions and email guidance were provided to doctors, while general emails targeted nursing staff.

**Table 1:** Large-volume paracentesis insertion checklist (based on NatSSIPs 2 and adapted from BSG safety checklist) IV: intravenous; HAS: human albumin solution; Kgs: kilograms; MCS: microscopy, culture, and sensitivity; NatSSIPs: National Safety Standards for Invasive Procedures 2

Consent, Procedural Verification, Site Marking
Drain inserted by:
Designation and bleep number:
Indication?
Consent obtained?
Confirmed presence of ascites?
Allergies?
Team Debriefs
IV access in situ and observations checked?
HAS prescribed and available?
Checked platelet count and clotting profile?
Pre-drain weight (in Kgs)?
Sign In, Time Out
Identity confirmed?
Time of drain insertion?
Site and type of drain inserted?
Local anaesthetic used and how much?
Dressing applied and left on free drainage?
Colour of the initial ascitic fluid drained?
Ascitic fluid samples taken and sent for MCS?
Sign Out
Any immediate complications?
Time for drain removal (maximum of 6 hours)?
HAS replacement instructions?
Diuretics reviewed?
Handover (Nursing Team to Complete)
Time when the drain was removed?
Any drain site pain or haematoma?
Colour of the ascitic drain?
Total HAS given?
Observations checked after drain removal?
Post-drain weight?

Cycle 2 (December 2023-March 2024)

A re-audit was performed, and the checklist’s usability on the CRS was refined based on verbal feedback from doctors and nurses. Logistical delays were addressed with the information technology team to create a more centralised system. Posters outlining initial data and procedural steps were displayed on wards, particularly in the drain clinic room. Additional training sessions for the nursing team were organised weekly in small groups.

Cycle 3 (April-July 2024)

Sustained reinforcement measures were implemented, including reminder emails, posters, and introductory slides during doctors’ induction sessions. A final re-audit was conducted to evaluate compliance.

Statistical analysis

Compliance data across the three PDSA cycles were analysed to determine significant differences using the Chi-Square Test for Independence. This statistical method tested the null hypothesis that compliance proportions remained consistent across cycles for each documentation category. Analysis was conducted using Python software, with a significance level of 0.05. P-values below this threshold indicated statistically significant improvements in compliance.

Ethical considerations

The study adhered to ethical guidelines and received approval from the Clinical Effectiveness Unit (CEU) in accordance with local hospital policies including confidentiality and data protection. No patient-identifiable information was used.

## Results

The results are organised into three documentation categories: pre-procedure documentation by doctors, post-procedure documentation by doctors, and post-procedure documentation by nurses. Compliance rates and their corresponding p-values, derived from the Chi-Square Test for Independence are summarised in Table 2.

**Table 2:** Compliance rates and their corresponding p-values, derived from the Chi-Square Test for Independence. ID: identification; IV: intravenous; HAS: human albumin solution; WCC: white cell count; MCS: microscopy, culture, and sensitivity

Category	Cycle 1 (n = 35)	Cycle 2 (n = 34)	Cycle 3 (n = 35)	p-value
HAS ordered and available	35	33	35	0.354
Confirmed presence of ascites	35	34	35	N/A
Patient ID and consent	33	33	28	0.035
IV access in situ	23	25	16	0.049
Pre-drain weight	8	19	15	0.019
Baseline observations	7	26	15	0.024
Checked clotting profile results	3	26	15	0.011
Checked platelet count	2	26	15	0.008
Plan for HAS replacement	34	34	35	0.785
Specimens sent for WCC + ascitic MCS	33	32	29	0.563
Time of drain insertion and removal	30	32	35	0.354
Ascitic fluid initial colour	26	33	33	0.257
Notes on diuretics	1	1	17	0.002
Post-procedure observations	25	23	25	0.725
Drain output and amount of HAS given	22	24	31	0.094
Colour of ascitic fluid	11	10	21	0.030
Post-drain weight	7	14	17	0.042
Drain site pain/haematoma monitored	0	0	21	0.000

Pre-procedure documentation by doctors

**Figure 1:**
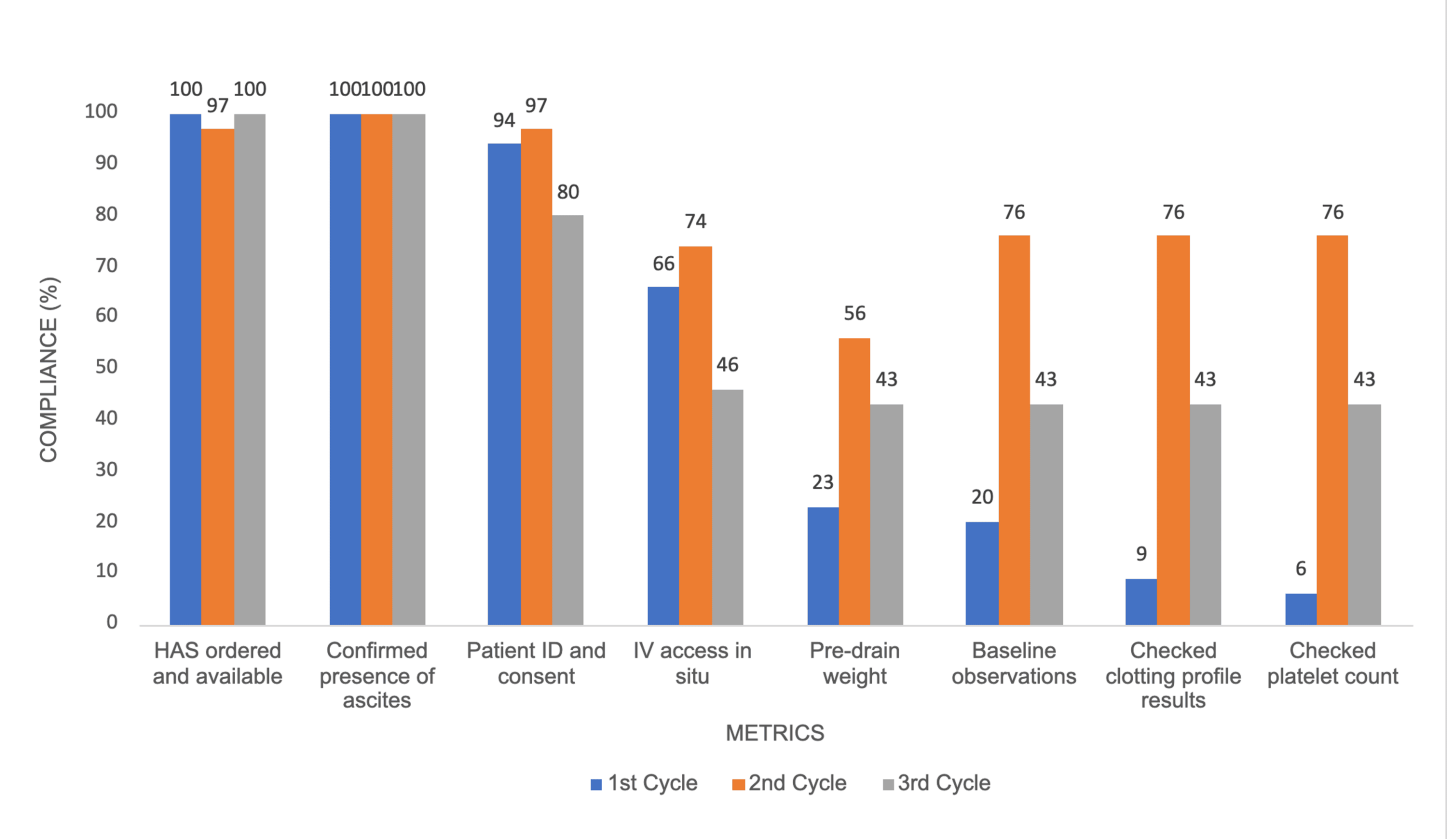
Graph showing pre-procedure documentation compliance by doctors across three cycles HAS: human albumin solution

Metrics such as ‘HAS ordered and available’ and ‘Confirmed presence of ascites’ maintained consistently high compliance across all cycles. ‘Patient identification (ID) and consent’ showed a compliance decrease from 33/35 (94%) in Cycle 1 to 28/35 (80%) in Cycle 3, with a significant p-value of 0.035. Other metrics demonstrated significant differences in compliance across the cycles. Compliance for ‘Pre-drain weight’ increased from 8/35 (23%) in Cycle 1 to 19/34 (56%) in Cycle 2 but dropped to 15/35 (43%) in Cycle 3 (p = 0.019). ‘Baseline observations’ rose from 7/35 (20%) in Cycle 1 to 26/34 (76%) in Cycle 2 and decreased to 15/35 (43%) in Cycle 3 (p = 0.024). Similarly, ‘Checked clotting profile results’ and ‘Checked platelet count’ showed significant differences with p-values of 0.011 and 0.008, respectively (Figure 1; Table 2).

Post-procedure documentation by doctors

**Figure 2:**
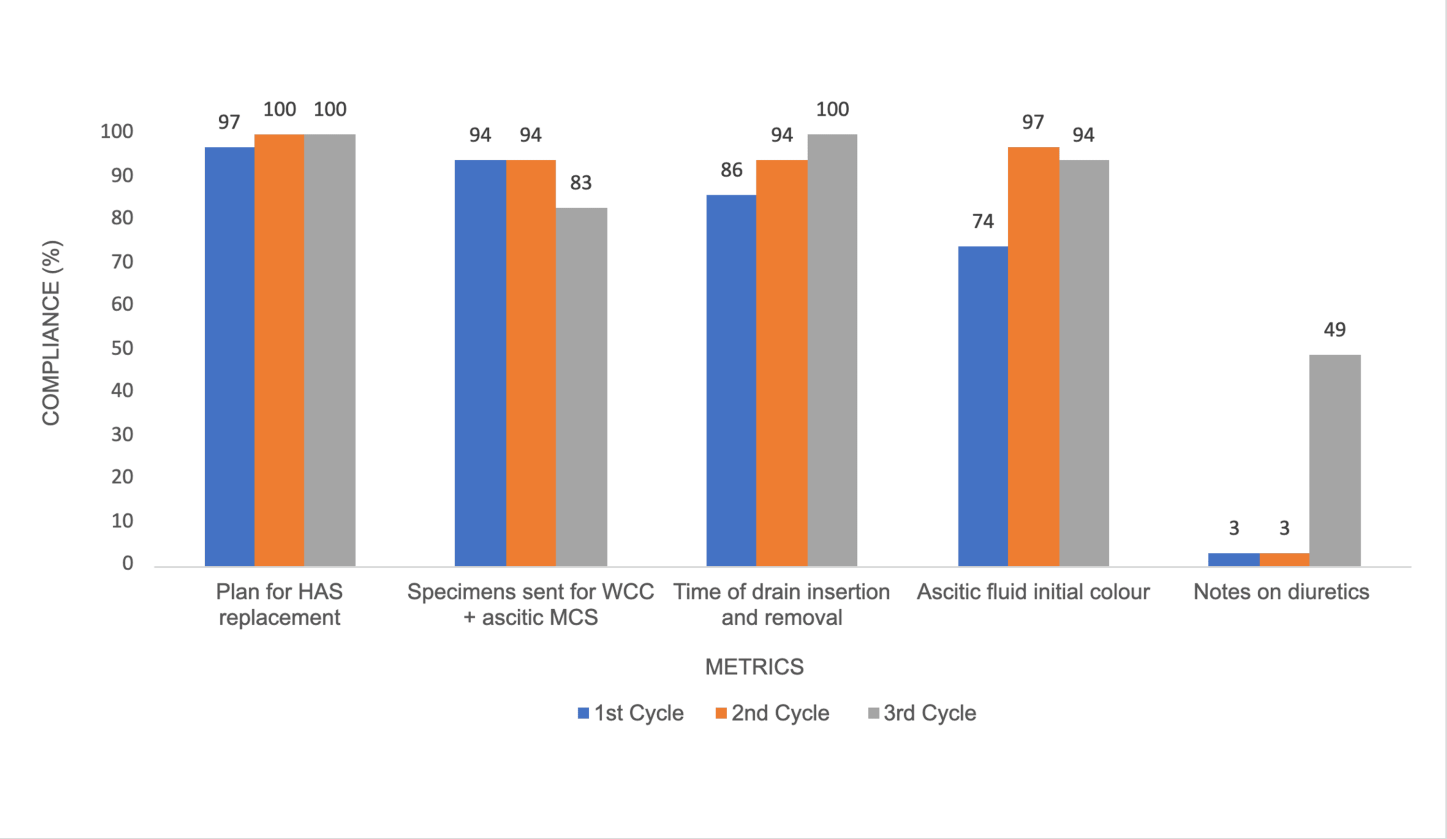
Graph showing post-procedure documentation compliance by doctors across three cycles HAS: human albumin solution

Compliance for ‘Plan for HAS replacement’ remained consistently high across all cycles (p = 0.785). Similarly, ‘Specimens sent for white cell count (WCC) + ascitic microscopy, culture, and sensitivity (MCS)’, ‘Time of drain insertion and removal’, and ‘Ascitic fluid initial colour’ showed no significant changes, with p-values of 0.563, 0.354, and 0.257, respectively. ‘Notes on diuretics’ showed a significant improvement, with compliance increasing from 1/35 (3%) in Cycle 1 to 17/35 (49%) in Cycle 3 (p = 0.002) (Figure 2; Table 2).

Post-procedure documentation by nurses

**Figure 3:**
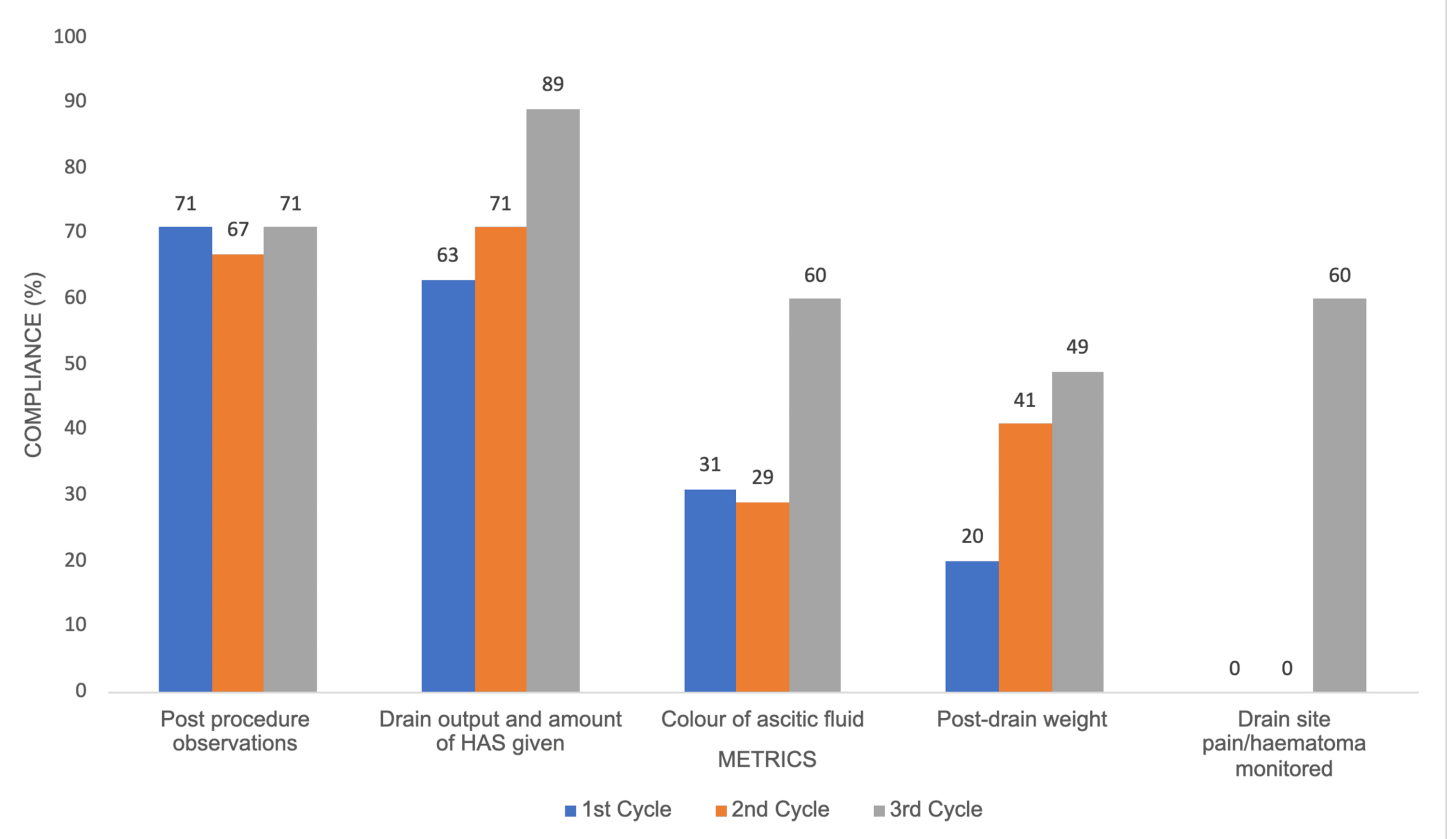
Graph showing post-procedure documentation compliance by nurses across three cycles HAS: human albumin solution

‘Post-procedure observations’ showed no significant change, with compliance rates of 25/35 (71%) in Cycle 1, 23/34 (68%) in Cycle 2, and 25/35 (71%) in Cycle 3 (p = 0.725). Significant improvements were observed in ‘Post-drain weight’ (p = 0.042) and ‘Colour of ascitic fluid’ (p = 0.030). Compliance for ‘Post-drain weight’ increased from 7/35 (20%) in Cycle 1 to 17/35 (49%) in Cycle 3. ‘Colour of ascitic fluid’ improved from 11/35 (31%) in Cycle 1 to 21/35 (60%) in Cycle 3. The most significant improvement was noted in ‘Drain site pain/haematoma monitored’, which rose from 0/35 (0%) in Cycles 1 and 2 to 21/35 (60%) in Cycle 3, with p < 0.001. ‘Drain output and amount of HAS given’ showed an increase in compliance, though the change was not statistically significant (p = 0.094) (Figure 3; Table 2).

## Discussion

The standardisation of LVP documentation through the use of a structured checklist and multi-faceted interventions resulted in significant improvements in documentation quality. This project underscores the critical importance of LocSSIPs, as highlighted by multiple studies. The iterative application of the PDSA cycle facilitated measurable enhancements in both pre- and post-procedure documentation metrics. Improved documentation has been widely shown to enhance patient safety and compliance across diverse clinical domains. Consistent with our findings, Hoshen and Mahmood demonstrated that standardised documentation processes improve patient outcomes by ensuring continuity of care and reducing the risk of complications [7].

The engagement of a multidisciplinary team was instrumental in driving these improvements. Collaborative approaches, as summarised by Tagar et al., are integral to the development and implementation of LocSSIPs, ensuring procedural safety [8]. Furthermore, Bamford and Smith emphasised the importance of customising LocSSIPs to align with workflow-specific needs, such as those in intensive care, thereby promoting adaptability and long-term adoption [9].

In our study, compliance metrics such as ‘Checked clotting profile results’ and ‘Checked platelet count’ showed significant improvement during Cycle 2 and this could mean there is a reduced procedure complication. This finding aligns with those of Hache et al., who utilised similar checklist-based approaches to reduce procedural complications [10]. Similarly, our results corresponded with Hessey et al.’s identification of gaps in pre-procedure blood test documentation and albumin administration areas requiring targeted intervention in our study as well [11]. In contrast, significant improvements in albumin prescription and management were noted by Fyson et al. [12]. Variability in compliance for metrics such as ‘Patient ID and consent’, ‘Pre-drain weight’, and ‘Baseline observations’ highlighted the need for ongoing education and system refinements. This finding resonates with Liew and Beech who reported that sustaining checklist adherence necessitates periodic training and reminders-key elements of our intervention strategy [13]. Education played a pivotal role in reinforcing checklist adherence. Mason et al., for example, demonstrated the effectiveness of training in improving compliance during endoscopy procedures [14]. Similar to our study, Tagar et al. observed compliance issues in follow-up audits six months post-implementation [8].

Our findings align with broader literature emphasising the transformative role of electronic health records (EHRs) in improving care quality across multiple dimensions. EHR systems streamline workflows, standardise processes, and minimise errors, thereby enhancing documentation quality and patient outcomes. A study by McCarthy et al. illustrates how EHR interventions via structured forms and automated prompts improve documentation practices by reducing cognitive workload and allowing clinicians to focus on patient care [15]. Additionally, systematic reviews by Chaudhry et al. and Buntin et al. provide compelling evidence of the positive impact of health information technology (HIT) on healthcare delivery. They demonstrate that electronic systems not only enhance care quality but also streamline administrative processes and reduce medical errors [16,17].

Finally, by integrating our LVP documentation checklist into the CRS, we improved accessibility and enabled real-time data recording. Further validation of this approach comes from Kyaw et al., who emphasised embedding LocSSIPs into electronic systems to enhance accessibility and usability [18]. Yuan et al. similarly demonstrated that structured digital templates significantly improve post-procedure documentation and patient safety in ascitic paracentesis [19]. While physical stickers, as utilised by Hessey et al., remain a viable alternative, the cumulative evidence strongly supports the adoption of electronic proformas for improving procedural documentation [11]. The integration of our checklist into the CRS provided a streamlined solution, further reinforcing the utility of digital templates in minimising omissions and ensuring systematic documentation. Such initiatives are essential for optimising patient outcomes, particularly in procedures like LVP, where consistent and comprehensive documentation is critical.

Study limitations

This study had several limitations. First, the sample size was relatively small, with 35, 34, and 35 procedures across the three cycles. The exclusion of LVPs performed in departments such as intensive care, surgical units, and acute medical units further limited the generalisability of the findings. Additionally, as a single-centre study, the results may not reflect broader trends across institutions. Finally, while improvements in documentation were observed, the direct impact on clinical outcomes was not measured. Instead, the study relied on existing literature linking enhanced documentation to improved procedural quality and patient safety.

## Conclusions

The implementation of a standardised documentation process for LVP procedures in a tertiary care hospital resulted in substantial improvements in the completeness and accuracy of procedural records. By integrating a LocSSIPs-based checklist into the CRS, the project not only enhanced accessibility but also promoted sustained adherence to documentation standards. The findings underscore the transformative potential of multidisciplinary collaboration, iterative quality improvement cycles, and targeted educational interventions in fostering procedural consistency and improving patient safety. This study highlights the critical role of structured checklists in mitigating procedural risks and promoting best practices, as supported by existing evidence across various medical disciplines. Despite its successes, the study also reveals the challenges of sustaining compliance, emphasising the need for continuous training, regular audits, and adaptive refinements.

Future efforts should focus on developing collaborative, cross-institutional frameworks that standardise procedural documentation and ensure scalability across diverse clinical settings. The integration of advanced digital tools, including artificial intelligence, could further enhance adherence and streamline workflows. Sustaining these improvements will require ongoing education, multidisciplinary engagement, and a commitment to embedding patient safety as a core component of procedural practice.
